# Letter from Dr. J. C. Clark

**Published:** 1843-06

**Authors:** C. J. Clark

**Affiliations:** Secretary Jacksonville Medical Board


### ARTICLE VI.

### LETTER *from Dr. J. C. Clark.*

*Jacksonville, Alabama, March 28th,* 1843.

MESSRS. EDITORS:—I have been requested by Dr. C. C. Porter, to furnish you with an outline of the "act" of the legislature of Alabama, "regulating the practice of dental surgery." In accordance with which I write the following:—The first, and I believe the only act on this subject, was passed at the session of 1841 and '42, and approved December 31st, 1841. The first section provides, that after the first Monday in December following, "it shall be the duty of each of the Medical Boards of this State, to examine and license applicants to practise dental surgery, under the same rules and regulations, and subject to the same restrictions as those who apply for license to practise medicine:" and, in order more fully to carry the act into effect, that "it shall be the duty of each of the Medical Boards, where the same is practicable, to add to their body, by election, a professional dentist, having the requisite qualifications, which dentist so added, shall constitute a part of the board. Section 2nd provides a penalty, which, for each offence is the forfeiture and payment of a sum not exceeding fifty dollars, recoverable before any court having jurisdiction of the same; one-half to the informer, the other to the county in which the suit is brought. Section 3d, makes all bonds, notes or promissory obligations or assumpsits, for services by a professional dentist, or in the line of professional dentistry, by an individual not having a license, "utterly void and of no effect," with a provision, that the prohibition shall not extend to persons licensed to practice medicine and surgery, or graduates of regularly constituted medical institutions in the United States.

The following physicians constitute the medical board at this place.—WM. WILLIAMSON, JAS. C. FRANCIS, A. PELHAM, G. R. GRANT, and C. J. CLARK.

In accordance with a provision of the above act, we elected C. C. Porter, surgeon dentist, a member of our board.

I may remark that examinations before medical boards have been conducted rather loosely in this state, and individuals have frequently practised medicine without paying any regard to the law requiring them to have licenses; though at this time public attention is directing itself more to the subject, and soi-disant "doctors" are finding it necessary to have their qualifications certified by those vested with the authority to do so. But it is the curse of "the freest country in the world" that the standard of medical education is so low. Since the commencement of the financial crisis that has not even yet terminated, hundreds of men—from him who has not yet reached his majority, up to the grey headed father—having been thrown out of business, or broken up, and finding easy access, have crowded into the professions; and however mortifying it may be, it is the fact, that the ill-educated more frequently resort to the medical profession; ignorance there finding an Achillean shield of brazen impudence, substituted for that knowledge attainable only by long application and perseverance. It is nothing uncommon for those who have scarcely the elements of an *English* education to launch into a course of professional reading, and after delving and floundering along for a *few* months, without having mastered even the technicalities, to say nothing of the host of facts, the sublime generalizations, and recondite principles of the science, or rather sciences, to go forth to battle with disease at the sacrifice of human happiness, and too frequently that of human life. And should one of these striplings, David-like, slay one giant, he is of course, a prodigy, possessing an *instinctive* knowledge of diseases and remedies far surpassing any thing to be obtained from *"books,"* or within the walls of a college, and must be crowned to the exclusion of him who, by laborious research and great sacrifice of time, money, and health, honestly and honourably qualifies himself for the important task he undertakes. Amongst other professions, that of dental surgery has had its host of ignorant practitioners. The vender of pills and mixer of boluses has abandoned his patients; the hopeful youngster of no business has *adopted* a profession; the carpenter relinquished his tools; the broken merchant his store and yard-stick; the engineer his machinery; the tailor his hissing goose; jumped from his counter, and *all*, armed with saw, file and scraper, have gone forth conquering and to conquer. It is this prostitution of the professions that has brought the travelling phrenologist, dentist, and, perhaps I might say mesmerist, to be looked upon something like the exhibitor of a puppet show, or the professor of legerdemain.

It is a noble undertaking to redeem dental surgery from such mercenary hands, and raise it to that high standing to which its importance so justly entitles it. It is a proud thing to rear a new temple to Esculapius, or to erect a new shrine to Minerva; and wishing you and your coadjutors eminent success in your laudable undertaking and praiseworthy labours, and asking pardon for a digression which has exceeded the main story of this letter, I am respectfully yours, &c. C. J. Clark, M. D.

*Secretary Jacksonville Medical Board,*

